# p38 MAPK activity is associated with the histological degree of interstitial fibrosis in IgA nephropathy patients

**DOI:** 10.1371/journal.pone.0213981

**Published:** 2019-03-21

**Authors:** Jeonghwan Lee, Jung Nam An, Jin Ho Hwang, Hajeong Lee, Jung Pyo Lee, Sung Gyun Kim

**Affiliations:** 1 Department of Internal Medicine, Hallym University Sacred Heart Hospital, Anyang, Gyeonggi-do, Korea; 2 Department of Internal Medicine, Seoul Metropolitan Government Seoul National University Boramae Medical Center, Seoul, Korea; 3 Department of Internal Medicine, Chung-Ang University Hospital, Seoul, Korea; 4 Department of Internal Medicine, Seoul National University Hospital, Seoul, Korea; Hopital Tenon, FRANCE

## Abstract

Activation of p38 mitogen-activated protein kinase (MAPK) is associated with tissue fibrosis, and inhibition of p38 MAPK can attenuate the progression of fibrosis. We aimed to investigate whether p38 MAPK activity in kidney tissue confirmed by immunohistochemical staining is associated with renal tubulointerstitial fibrosis in chronic kidney disease patients with IgA nephropathy. We collected kidney biopsy specimens from 341 IgA nephropathy patients and 15 control patients to identify the clinical and histopathological factors associated with kidney tubulointerstitial fibrosis and to find an association between kidney phosphorylated p38 immunoactivity and pathological grading. In addition, we aimed to investigate whether the anti-fibrotic effect of p38 MAPK inhibition can be identified by assessing the immunostaining intensity of phosphorylated p38 in kidney tissue. A renal tubulointerstitial fibrosis model was introduced using 7-week-old C57BL/6 mice subjected to unilateral ureteral obstruction (UUO). The p38 MAPK inhibitor SB-731445 was injected intraperitoneally every day for 7 days, and changes in renal fibrosis-associated markers were investigated. Assessment of kidney biopsy specimens from IgA nephropathy patients revealed that the degree of interstitial fibrosis was significantly associated with the tissue immunoactivity of phosphorylated p38. High-grade interstitial fibrosis was associated with a low glomerular filtration rate, high proteinuria, and high-grade histopathological changes, including tubular atrophy, interstitial inflammation, and glomerular sclerosis. In a mouse UUO model, renal protein expression of COL1 and phosphorylated p38 were significantly increased, and the protein expression of COL1 and phosphorylated p38 decreased in mice administered 10 mg/kg/day p38 MAPK inhibitor. We found that kidney interstitial fibrosis is associated with increased immunoactivity of phosphorylated p38 in a UUO mouse model and in human IgA nephropathy patients and that the anti-fibrotic effect of p38 MAPK inhibition can be confirmed using immunohistochemical staining for phosphorylated p38 in kidney tissue.

## Introduction

Chronic kidney disease is a pathological condition of decreased renal function with evidence of kidney injury characterized by tubulointerstitial fibrosis. Tubulointerstitial fibrosis is the final common pathway for most progressive kidney diseases and leads to advanced chronic kidney disease [1,2]. Fibrosis is not merely the final morphological feature of deteriorating chronic kidney disease but is rather an amalgamation of various pathologic processes involving the extracellular matrix, tissue proteases, tissue inflammation, fibroblasts, the tubular epithelium, and the microvasculature [3]. The discovery of a drug that can reduce renal fibrosis could be promising for delaying the progression of chronic kidney disease.

The p38 mitogen-activated protein kinase (MAPK) pathway is an intracellular signal transduction pathway involved in the production of proinflammatory and profibrotic mediators [4], and it is important in the pathogenesis of fibrosis with extracellular matrix synthesis. Upon activation of this pathway, p38 MAPK is phosphorylated, resulting in the sequential phosphorylation and activation of downstream kinases [5]. p38 MAPK is a signal transduction mediator that is expressed as four isoforms (α, β, γ, and δ), and of these, the α, β, and δ isoforms are predominant in the kidney [6,7]. Phosphorylation of p38α results in its translocation to the nucleus and the activation of transcription factors involved in the production of proinflammatory mediators and extracellular matrix proteins [8].

Activation of p38 MAPK has been demonstrated in fibrosis in various organs, including pulmonary fibrosis [9], peritoneal membrane fibrosis [10,11], and cardiac fibrosis [12]. Blockade of p38 MAPK has been shown to inhibit TGF-β1-induced collagen expression in fibroblasts, hepatic cells, and mesangial cells [13]. In animal models of sclerodermatous chronic graft-versus-host disease, blockade of p38 MAPK reduced skin fibrosis [14]. In addition, researchers have also found that p38 MAPK plays a role in the development of renal fibrosis [4,15–17]. However, most results from these studies were derived from specific animal models, and the effects of p38 MAPK inhibitors on the prevention or regression of kidney fibrosis were not fully validated. To date, no study has investigated the relationship between human kidney fibrosis and kidney p38 MAPK activity. In this study, we aimed to investigate whether p38 MAPK activity is associated with renal tubulointerstitial fibrosis in both an animal kidney fibrosis model and human IgA nephropathy and whether the anti-fibrotic effect of p38 MAPK inhibition can be confirmed using immunohistochemical staining for phosphorylated p38 in kidney tissue.

## Methods and materials

### IgA nephropathy, kidney fibrosis and phosphorylated p38 immunohistochemistry in patient specimens

We included 341 IgA nephropathy patients who underwent kidney biopsy at Seoul Metropolitan Government Seoul National University Boramae Medical Center and Seoul National University Hospital. Kidney biopsy was performed only for the clinical purpose of investigating the specific cause of suspicious intrinsic kidney disease among patients who showed unexplained proteinuria (with or without hematuria) or azotemia. Samples of urine, plasma, serum and biopsy tissue from all patients who underwent renal biopsy were prospectively collected with written informed consent before enrollment and biopsy for academic research. We collected data from 341 adult patients with biopsy-proven IgA nephropathy from January 2009 to December 2017. Only the first biopsy specimens were included in this study among patients who underwent repeated kidney biopsy more than 2 times. Data about demographic and clinical parameters, such as age, sex, body mass index (BMI), blood chemistry, spot urine protein-creatinine ratio (uPCR) and comorbidity (hypertension, DM and viral hepatitis), were collected at the time of kidney biopsy. Information regarding blood chemistry, uPCR and prescribed medications (angiotensin-converting enzyme inhibitors or angiotensin II receptor blockers and immunosuppressive agents) was recorded during the follow-up period. The estimated glomerular filtration rate (eGFR) was calculated using the isotope dilution mass spectrometry traceable 4-variable modified Modification of Diet in Renal Disease Study equation. Biopsy tissues were examined using light, electron and immunofluorescence microscopy, and the histologic diagnosis was made by a renal pathologist. Interstitial fibrosis and tubular atrophy and interstitial inflammation scores were graded as the percentage of the affected area as follows: 0 (none), 1 (mild, ≤ 25%), 2 (moderate, 26–50%) and 3 (severe, >50%). Information on fibrointimal thickening and hyaline arteriolosclerosis was obtained from the final pathologic record of each patient. To ensure accuracy of the histopathologic findings, 2 independent pathologists blinded to the original findings reviewed 30 random samples each. Kidney biopsy specimens from 57 randomly selected and approved patients were prepared for tissue immunohistochemical staining. In addition, we included 15 control patients who showed no specific pathologic changes in kidney biopsy specimens to compare kidney tissue p38 MAPK activity. Activity of p38 MAPK in kidney tissue was assessed using immunohistochemical staining for phosphorylated p38 among these randomly selected 57 IgA nephropathy patients. This study was approved by the ethics committee of the Institutional Review Board of Seoul Metropolitan Government Seoul National University Boramae Medical Center (H-10-2018-44), and all participants provided written informed consent before enrollment. All clinical investigations were conducted in accordance with the guidelines of the 2013 Declaration of Helsinki.

### Animal models of unilateral ureteral obstruction

The animal experiments were approved by the Seoul Metropolitan Government Seoul National University Boramae Medical Center Institutional Animal Care and Use Committee in accordance with the National Research Council ‘Guidelines for the Care and Use of Laboratory Animals’ (No. 2015–0004) and were conducted at the Seoul Metropolitan Government Seoul National University Boramae Medical Center animal laboratory under specific pathogen-free conditions. Seventeen male C57BL/6 mice (20–22 g, aged 7 weeks, purchased from KOATECH, Kyeonggi-do, Korea) were randomly assigned to 1 of 4 groups: sham-operated normal control without treatment (n = 2), unilateral ureteral obstruction (UUO) mice treated with either vehicle (20% DMSO) or p38 MAPK inhibitor at a dosages of 5 or 10 mg/kg/day (n = 5 in each group). The mice were anesthetized with ketamine (100 mg/kg body weight) and pentobarbital sodium (50 mg/kg body weight). The UUO operation was performed to induce kidney fibrosis according to the following protocol. The left flank of each mouse was opened with an incision, the left kidney and proximal ureter were exposed, the proximal ureter was ligated in two places with a 1–2 mm gap using a 4–0 silk, and the ureter was cut between the two knots. Sham surgery was performed in the same way as the UUO operation, but ureter ligation and cutting were not performed. All surgical procedures were performed on a heating pad to maintain a constant body temperature (40°C). The p38 MAPK inhibitor SB-731445 (GlaxoSmithKline Research & Development Limited, Brentford, United Kingdom, GSK) was diluted in 20% DMSO and injected intraperitoneally every 24 hours from just after completion of the UUO operation (postoperative day (POD) 0) until POD 6. The mice were all sacrificed at POD 7. The left kidneys of the mice were removed and cut in the longitudinal direction. One-half of the kidney was used for histological examination (both frozen tissue and paraformaldehyde fixation), and the other half was cut in the transverse direction to obtain tissue for Western blotting and real-time polymerase chain reaction (PCR). All animal experiments were repeated independently at least three times following the same protocol to assess significance.

### Histologic examination for fibrosis

When the kidneys were extracted from the mice, exsanguination was performed, and the extracted kidneys were fixed with 10% buffered formalin and embedded with paraffin. Paraffin-embedded kidney tissue blocks were cut into 4 μm thick sections and stained with Masson’s trichrome to evaluate the degree of fibrosis [18]. For each kidney section, at least 8 fields were randomly selected and photographed at a 50 × magnification (Olympus Imaging America, Center Valley, CA). The area of fibrosis and total tissue were measured using morphometric analysis software (Qwin 3, Leica, Mannheim, Germany) and the mean value of area ratio was used as the fibrosis index.

### Quantitative real-time PCR

Total RNA was extracted from the kidney tissue or cells using a RNeasy kit (Qiagen GmBH, Hilden, Germany), and mRNA concentrations were measured using quantitative real-time PCR. For each sample, 1 μg of RNA was reverse transcribed using oligo-d(T) primers and Avian Myeloblastosis Virus Reverse Transcriptase Taq polymerase (Promega, Madison, Wis., USA). Real-time PCR was performed using assay-on-demand TaqMan probes and primers for alpha smooth muscle actin, type I alpha 1 collagen, fibronectin, p38 MAPK, MCP-1, periostin, and glyceraldehyde 3-phosphate dehydrogenase (GAPDH; Applied Biosystems). Quantitative real-time PCR was carried out using an ABI PRISM 7500 Sequence Detection System (Applied Biosystems, Foster City, CA). The specific mRNA levels were normalized to those of GAPDH, as a reference gene, in the sample. Relative quantification was performed using the Δ ΔCT method [19].

### Western blotting analysis

Kidney tissues were lysed in RIPA buffer [50 mM Tris·HCl, pH 7.3; 150 mM NaCl; 0.1 mM EDTA; 1% (vol/vol) sodium deoxycholate; 1% (vol/vol) Triton X-100; 0.2% NaF; and 100 μM Na_3_VO4] supplemented with complete protease inhibitors (Roche Applied Science, Indianapolis, IN). The kidney homogenate was centrifuged at 12,000 g for 30 min at 4°C, and the protein concentration of the supernatant was determined by the Bradford method. Equal amounts (80 μg) of extracted proteins were separated by 10% sodium dodecyl sulfate–polyacrylamide gel electrophoresis (SDS-PAGE) and transferred onto Immobilon-FL 0.4 μM polyvinylidene difluoride membranes (Millipore, Bedford, MA, USA). Tris-buffered saline containing 0.1% Tween 20 was used as the washing buffer. Primary antibodies against αSMA (Abcam), Collagen-1 (Abcam), phosphorylated p38 (Cell Signaling Technology), and β-actin (Sigma-Aldrich, St. Louis, MO, USA) were used for immunoblotting. After probing with primary antibodies, anti-rabbit IgG (1:5,000; Cell Signaling Technology, Danvers, MA) and anti-mouse (1:6,000 for β-actin; Cell Signaling Technology) secondary antibodies were used. Detection of the labeled proteins was performed by an enhanced chemiluminescence system (ECLTM PRN 2106; Amersham Pharmacia Biotech, Buckinghamshire, UK), and the band intensities were analyzed using a gel documentation system (Bio-Rad Gel Doc 1000 and Multi-Analyst version 1.1). The antibodies used for Western blotting and immunohistochemistry are listed in S1 Table.

### Immunohistochemistry

We performed immunohistochemical staining for phosphorylated p38 on kidney tissues from 57 IgA nephropathy participants who had unstained paraffin slides and all mouse kidney tissues. Kidney sections were deparaffinized and rehydrated with xylene and ethanol. Endogenous streptavidin activity was blocked by 0.3% hydrogen peroxide. Antigens were retrieved by heating paraffin-embedded sections in 10% citrate buffer in a microwave oven, 5 times for 5 min per session. The sections were probed with primary antibodies, followed by counterstaining with Mayer’s hematoxylin (Sigma-Aldrich). The degree of nonspecific staining was assessed using secondary antibodies and isotype control IgGs. The stained slides were viewed on an Olympus inverted microscope (Olympus Imaging America, Center Valley, CA), and at least 8 fields (×100 magnification) for each kidney specimen were photographed under a light microscope. The p38 MAPK-positive area (%) was evaluated by quantitative immunohistochemical staining analysis using ImageJ (ImageJ version 1.52d, Wayne Rasband, National Institute of Health, USA). The mean value of the phosphorylated p38 MAPK-positive tissue area (%) was compared between groups according to the degree of kidney fibrosis.

### Cell culture and TGF-β challenge combined with a p38 MAPK inhibitor

Primary human tubular epithelial cells (hTECs) were isolated from the noncancerous part of the human kidney tissue obtained from kidneys that were surgically removed due to renal cell carcinoma. hTECs were cultured for four to six passages in EGM-2 MV medium (Clonetics Co., San Diego, CA, USA) supplemented with 10% fetal bovine serum, 1 mg/mL hydrocortisone, 12 mg/mL bovine brain extract, 50 mg/mL gentamycin, 50 ng/mL amphotericin B, and 10 ng/mL epidermal growth factor. The cells were then stimulated with human recombinant TGF-β (2 ng/ml; PeproTech, Rocky Hill, NJ, USA) in the presence or absence of different concentrations (2–4 μΜ) of SB-731445. Microscopic morphological changes were observed between the groups. Quantitative real-time PCR for fibrosis markers, including collagen-1, fibronectin, and periostin, was carried out using an ABI PRISM 7500 Sequence Detection System (Applied Biosystems, Foster City, CA). All *in vitro* experiments were repeated 3 times to confirm the reproducibility.

### Statistical analysis

Physiological and laboratory data are expressed as the means ± SD. Statistical analysis was carried out using IBM SPSS 20.0 for data from IgA nephropathy patients and GraphPad Prism 7.0 (GraphPad Software, San Diego, CA) in the animal and cell experiments. In the analysis of human IgA nephropathy patients, differences between groups were tested with ANOVA and chi-square analysis, and differences between groups in the animal experiments were analyzed using the nonparametric Mann-Whitney U test. When comparing more than two groups, we used a nonparametric Kruskal-Wallis test. A value of *P* < 0.05 was considered to indicate a statistically significant difference.

## Results

### Relationship between the degree of fibrosis and the activity of p38 MAPK in a human IgA nephropathy cohort

**Fig 1:**
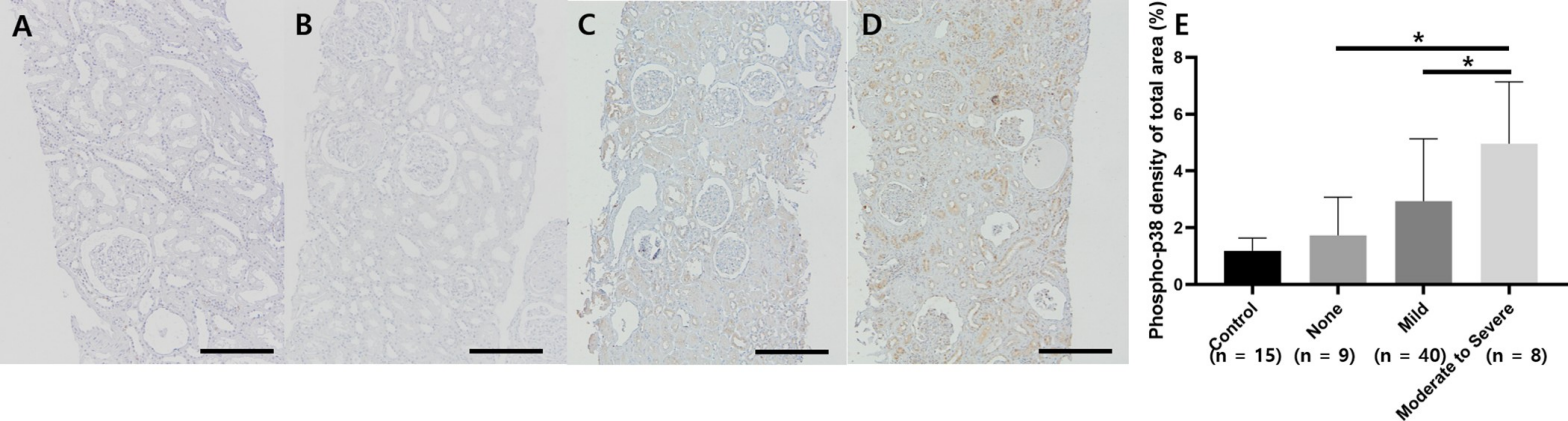
Immunoactivity of phosphorylated p38 in IgA nephropathy patients according to the grade of interstitial fibrosis (original magnification x100). Control panel shows the phosphorylated p38 kidney tissue activity among patients who have no pathologic changes in kidney biopsy specimens. (Fig 1-A) The immunoactivity of phosphorylated p38 in tissues from IgA nephropathy patients with moderate interstitial fibrosis (Fig 1-D) was higher than that in tissues from patients with no interstitial fibrosis (Fig 1-B) or mild interstitial fibrosis (Fig 1-C). The immunoactivity of phosphorylated p38 in patients with a high degree of interstitial fibrosis was higher than immunoactivity in those with a low degree of kidney interstitial fibrosis (Fig 1-E; overall *P* < 0.001; control vs. none, *P* = 0.588; none vs. moderate to severe, *P* = 0.001; mild vs. moderate to severe, *P* = 0.021).

We investigated whether the activity of p38 MAPK is increased in human IgA nephropathy cohort patients and whether it is associated with the degree of kidney fibrosis. Table 1 summarizes the clinical characteristics of participants according to the degree of kidney interstitial fibrosis. IgA nephropathy patients with high-grade interstitial fibrosis showed higher age, high blood pressure, high levels of serum creatinine, a low glomerular filtration rate, high proteinuria, and high prevalence of statin use. The other clinical parameters, including sex, BMI, comorbidities, serum IgA levels, microscopic hematuria, were all comparable between groups. Fig 1 shows the immunohistochemical staining for phosphorylated p38 in 57 IgA nephropathy patients and 15 control patients according to the grade of interstitial fibrosis. The mean age, serum creatinine levels, and uPCR of control patients was 22 ± 6.4 years old (18–38), 1.12 ± 0.32 mg/dl (0.64–1.88), and 0.18 ± 0.26 mg/mg (0.03–0.88), respectively. The expression of phosphorylated p38 was mainly located in the kidney tubules and interstitium and was more prominent in patients with higher degrees of interstitial fibrosis. The immunoactivity of phosphorylated p38 in patients with a high degree of interstitial fibrosis was higher than the immunoactivity in those with a low degree of kidney interstitial fibrosis (Fig 1, overall *P* < 0.001).

**Table 1:** Clinical characteristics of participants according to the degree of interstitial fibrosis.

Characteristics		Interstitial fibrosis			*P* value
	Total (n = 341)	None (n = 70)	Mild (n = 220)	Moderate to severe (n = 51)	
Age, years	40.2 ± 15.2	36.7 ± 16.6	40.4 ± 15.1	43.9 ± 13.2	0.032
Gender, male, %	170 (49.9%)	39 (55.7%)	108 (49.1%)	23 (45.1%)	0.478
BMI, kg/m^2^	22.6 ± 3.1	21.9 ± 3.2	22.8 ± 3.0	22.2 ± 3.1	0.381
Systolic BP, mm Hg	122.4 ± 15.7	118.5 ± 13.7	122.5 ± 16.1	127.2 ± 15.1	0.011
Diastolic BP, mm Hg	77.0 ± 11.8	73.9 ± 9.3	77.0 ± 12.2	81.10 ± 11.8	0.005
Comorbidity, numbers (%)					
Diabetes mellitus	15 (4.4%)	1 (1.4%)	12 (5.6%)	2 (3.9%)	0.345
Hypertension	199 (58.4%)	36 (51.4%)	128 (58.7%)	35 (68.6%)	0.165
HBV infection	12 (3.5%)	1 (1.7%)	10 (5.0%)	1 (2.0%)	0.390
HCV infection	1 (0.3%)	0 (0.0%)	1 (0.5%)	0 (0.0%)	0.760
Serum IgA level, mg/dl	309.3 ± 103.4	308.3 ± 100.2	306.3 ± 102.4	322.7 ± 112.4	0.623
Serum creatinine, mg/dl	1.27 ± 1.18	1.29 ± 1.92	1.12 ± 0.54	1.93 ± 1.60	< 0.001
IDMS-MDRD eGFR, ml/min/1.73 m^2^	74.4 ± 32.3	90.0 ± 34.2	76.2 ± 29.0	45.3 ± 24.2	< 0.001
Serum albumin, g/dl	3.8 ± 0.5	3.9 ± 0.5	3.9 ± 0.5	3.7 ± 0.5	0.021
Cholesterol, mg/dl	187.9 ± 38.2	179.3 ± 33.5	188.5 ± 38.6	197.8 ± 41.3	0.030
Uric acid, mg/dl	6.1 ± 1.7	5.6 ± 1.5	6.0 ± 1.6	7.1 ± 1.8	< 0.001
hs-CRP, mg/dl	0.28 ± 0.68	0.24 ± 0.45	0.32 ± 0.80	0.20 ± 0.28	0.476
Spot urine protein/Cr, mg/mg	1.76 ± 2.30	1.38 ± 2.40	1.60 ± 2.17	2.92 ± 2.40	< 0.001
Microscopic hematuria, numbers (%)	170/196 (86.9%)	27/32 (84.4%)	117/135 (86.7%)	26/29 (89.7%)	0.831
Treated with RAS blockade (ACEi/ARB), numbers (%)	196/339 (57.8%)	34/70 (48.6%)	129/218 (59.2%)	33/51 (64.7%)	0.164
Treated with statin, numbers (%)	69/216 (31.9%)	7/37 (18.9%)	43/145 (29.7%)	19/34 (55.9%)	0.002
Treated with immunosuppressive agents, numbers (%)	46/339 (13.6%)	4/70 (5.7%)	31/218 (14.2%)	11/51 (21.6%)	0.038
Final serum creatinine, mg/dl	1.52 ± 1.61	1.29 ± 1.54	1.31 ± 1.20	2.75 ± 2.52	< 0.001
Final IDMS-MDRD eGFR, ml/min/1.73 m^2^	68.8 ± 32.3	82.3 ± 31.9	72.0 ± 29.4	36.6 ± 23.7	< 0.001
Final spot urine protein/Cr, mg/mg	1.01 ± 1.15	0.59 ± 0.66	0.92 ± 1.04	1.96 ± 1.04	< 0.001
End-stage renal disease, numbers (%)	16 (4.7%)	2 (2.9%)	7 (3.2%)	7 (13.7%)	0.004

Abbreviations: Cr, creatinine; BMI, body mass index; BP, blood pressure; HBV, hepatitis B virus; HCV, hepatitis C virus; hs-CRP, high-sensitivity C-reactive protein; RAS, renin-angiotensin-aldosterone system

Table 2 summarizes the histopathological findings according to the degree of interstitial fibrosis. In IgA nephropathy patients, high-grade fibrosis was associated with poor histopathological findings including tubular atrophy, interstitial inflammation, and glomerular sclerosis. Clinical characteristic comparisons based on the phosphorylated p38 activity in the kidney tissue are shown in Table 3. The clinical characteristics of 57 patients who underwent immunohistochemical staining for phosphorylated p38 and those of the other 284 patients were comparable, and no significant differences were found between the two groups (S2 Table). The degree of phosphorylated p38 immunohistochemical activity was categorized by the median value into upper (high expression group) and lower (low expression group) halves. Patients with high p38 MAPK activity showed a low glomerular filtration rate, high prevalence of microscopic hematuria, and high prevalence of immunosuppressive agent treatment. Table 4 shows the histopathological findings according to the degree of phosphorylated p38 immunohistochemical activity. In IgA patients, high phosphorylated p38 tissue expression levels were associated with high degrees of interstitial fibrosis (*P* < 0.001), interstitial inflammation (*P* = 0.001), and tubular atrophy (*P* = 0.005); vascular fibrointimal thickening (*P* = 0.024); and glomerular global sclerosis (*P* = 0.006). Vascular hyalinosis and arteriosclerosis were not associated with phosphorylated p38 tissue expression. Moreover, the degree of glomerular segmental sclerosis and crescent formation were also not associated with phosphorylated p38 immunoactivity. High phosphorylated p38 expression levels were correlated with a high degree of Hass IgA classification (*P* = 0.001).

**Table 2:** Histopathological characteristics of participants according to the degree of interstitial fibrosis.

Characteristics		Interstitial fibrosis			*P* value
	Total (n = 341)	None (n = 70)	Mild (n = 220)	Moderate to severe (n = 51)	
Tubular atrophy (n = 340)	(n = 340)	(n = 69)	(n = 220)	(n = 51)	< 0.001
None	55 (16.1%)	46 (66.7%)	5 (2.3%)	4 (7.8%)	
Mild	218 (63.9%)	22 (31.9%)	193 (87.7%)	3 (5.9%)	
Moderate	61 (17.9%)	1 (1.4%)	21 (9.5%)	39 (76.5%)	
Severe	6 (1.8%)	0 (0.0%)	1 (0.5%)	5 (9.8%)	
Interstitial inflammation (n = 337)	(n = 337)	(n = 70)	(n = 217)	(n = 50)	< 0.001
None	67 (19.6%)	40 (57.1%)	23 (10.6%)	4 (8.0%)	
Mild	216 (63.3%)	28 (40.0%)	172 (79.3%)	16 (32.0%)	
Moderate	52 (15.2%)	2 (2.9%)	21 (9.7%)	29 (58.0%)	
Severe	2 (0.6%)	0 (0.0%)	1 (1.3%)	1 (0.3%)	
Vessel, %					
Fibrointimal thickening	151 (44.3%)	20 (28.6%)	90 (40.9%)	41 (80.4%)	< 0.001
Hyalinosis	46 (13.5%)	2 (2.9%)	30 (13.6%)	14 (27.5%)	< 0.001
Arteriosclerosis	25 (7.3%)	1 (1.4%)	17 (7.7%)	7 (13.7%)	0.035
Segmental sclerosis (mean of %)	6.4 ± 8.1	3.6 ± 5.8	6.2 ± 8.1	11.2 ± 9.0	< 0.001
Global sclerosis (mean of %)	20.6 ± 21.9	11.8 ± 16.6	16.8 ± 17.4	48.8 ± 24.1	< 0.001
Crescent (mean of %)	1.4 ± 4.4	1.2 ± 3.3	1.4 ± 4.1	1.6 ± 6.5	0.911
Mesangial hypercellularity	329 (96.5%)	66 (94.3%)	214 (97.3%)	49 (98.0%)	0.407
Pathologic classification, %					
WHO (n = 213)	(n = 213)	(n = 41)	(n = 140)	(n = 32)	< 0.001
Class I	25 (11.7%)	11 (26.8%)	14 (10.0%)	0 (0.0%)	
Class II	93 (43.7%)	20 (48.8%)	68 (48.6%)	5 (15.6%)	
Class III	54 (25.4%)	8 (19.5%)	39 (27.9%)	7 (21.9%)	
Class IV	29 (13.6%)	1 (2.4%)	17 (12.1%)	11 (34.4%)	
Class V	12 (5.6%)	1 (2.4%)	2 (1.4%)	9 (28.1%)	
Lee SMK (n = 310)	(n = 310)	(n = 65)	(n = 198)	(n = 47)	< 0.001
Class I	8 (2.6%)	1 (1.5%)	7 (3.5%)	0 (0.0%)	
Class II	98 (31.6%)	32 (49.2%)	63 (31.8%)	3 (6.4%)	
Class III	143 (46.1%)	27 (41.5%)	101 (51.0%)	15 (31.9%)	
Class IV	45 (14.5%)	3 (4.6%)	24 (12.1%)	18 (38.3%)	
Class V	16 (5.2%)	2 (3.1%)	3 (1.5%)	11 (23.4%)	
Hass (n = 308)	(n = 308)	(n = 65)	(n = 196)	(n = 47)	< 0.001
Class I	9 (2.9%)	3 (4.6%)	6 (3.1%)	0 (0.0%)	
Class II	15 (4.9%)	1 (1.5%)	14 (7.1%)	0 (0.0%)	
Class III	84 (27.3%)	31 (47.7%)	50 (25.5%)	3 (6.4%)	
Class IV	160 (51.9%)	25 (38.5%)	118 (60.2%)	17 (36.2%)	
Class V	40 (13.0%)	5 (7.7%)	8 (4.1%)	27 (57.4%)	

**Table 3:** Clinical characteristics according to the degree of phospho-p38 immunohistochemical staining of kidney biopsy specimen from IgA nephropathy subjects.

Characteristics	Semiquantitative phosphorylated p38 expression		*P* value
	Low expression group (n = 29)	High expression group (n = 28)	
Age, years	40.3 ± 16.4	43.9 ± 15.1	0.395
Gender, male, %	12 (41.4%)	13 (46.4%)	0.701
BMI, kg/m^2^	21.3 ± 2.6	22.0 ± 2.3	0.365
Smoking	5 (17.2%)	5 (18.5%)	1.000
Systolic BP, mm Hg	122.1 ± 12.5	125.5 ± 16.1	0.386
Diastolic BP, mm Hg	77.2 ±10.0	77.6 ± 11.4	0.874
Comorbidity, (%)			
DM	0 (0.0%)	2 (7.4%)	0.228
HTN	13 (44.8%)	18 (66.7%)	0.100
HBV infection	2 (6.9%)	2 (7.4%)	0.941
HCV infection	1 (3.4%)	0 (0.0%)	0.330
Serum IgA level, mg/dl	300.4 ± 80.3	287.5 ± 81.7	0.569
Serum creatinine, mg/dl	1.09 ± 0.33	1.46 ± 0.53	0.002
IDMS-MDRD eGFR, ml/min/1.73 m^2^	80.6 ± 28.0	49.6 ± 20.6	< 0.001
Serum albumin, g/dl	3.9 ± 0.6	3.9 ± 0.6	0.898
Cholesterol, mg/dl	188.1 ± 35.2	183.4 ± 27.6	0.583
Uric acid, mg/dl	6.4 ± 1.3	6.1 ± 1.6	0.384
Spot urine protein/Cr, mg/mg	1.42 ± 1.30	2.15 ± 2.72	0.208
Microscopic hematuria	19/26 (73.1%)	26/27 (96.3%)	0.018
Final serum creatinine, mg/dl	1.21 ± 0.38	1.49 ± 0.59	0.040
Final IDMS-MDRD eGFR, ml/min/1.73 m^2^	78.0 ± 31.4	51.0 ± 27.5	< 0.001
Final spot urine protein/Cr, mg/mg	1.02 ± 1.03	1.39 ± 1.62	0.328
Treated with RAS blockade (ACEi/ARB), %	17 (58.6%)	17 (63.0%)	0.740
Treated with statin, %	7 (26.9%)	9 (33.3%)	0.611
Treated with immunosuppressive agents, %	0 (0.0%)	7 (25.9%)	0.003

Abbreviations: Cr, creatinine; BMI, body mass index; BP, blood pressure; HBV, hepatitis B virus; HCV, hepatitis C virus; hs-CRP, high-sensitivity C-reactive protein; RAS, renin-angiotensin-aldosterone system

**Table 4:** Histopathological findings according to the degree of phospho-p38 immunohistochemical staining of kidney biopsy specimens from IgA nephropathy subjects.

Histopathological findings	Semiquantitative phosphorylated p38 expression		*P* value
	Low expression group (n = 29)	High expression group (n = 28)	
Interstitial fibrosis			< 0.001
None	9 (31.0%)	0 (0.0%)	
Mild	20 (69.0%)	20 (71.4%)	
Moderate to severe	0 (0.0%)	8 (28.6%)	
Interstitial inflammation			0.001
None	7 (24.1%)	0 (0.0%)	
Mild	21 (72.4%)	17 (60.7%)	
Moderate to severe	1 (3.4%)	11 (39.3%)	
Tubular atrophy			0.005
None	3 (10.3%)	0 (0.0%)	
Mild	25 (86.2%)	17 (60.7%)	
Moderate to severe	1 (3.4%)	11 (39.3%)	
Vessel, %			
Fibrointimal thickening	10 (34.5%)	18 (64.3%)	0.024
Hyalinosis	2 (6.9%)	3 (10.7%)	0.610
Arteriosclerosis	2 (6.9%)	2 (7.1%)	0.971
Segmental sclerosis (mean of %)	6.6 ± 9.4	7.4 ± 6.9	0.720
Global sclerosis (mean of %)	12.4 ± 11.8	28.8 ± 27.3	0.006
Crescent (mean of %)	0.5 ± 1.3	3.1 ± 9.0	0.142
Pathologic classification, %			
WHO	(n = 20)	(n = 16)	0.381
Class I	3 (15.0%)	0 (0%)	
Class II	10 (50.0%)	9 (56.3%)	
Class III	5 (25.0%)	6 (37.5%)	
Class IV	2 (10.0%)	1 (6.3%)	
Class V	0 (0%)	0 (0%)	
Lee SMK	(n = 28)	(n = 21)	0.007
Class I	0 (0%)	0 (0%)	
Class II	17 (60.7%)	3 (14.3%)	
Class III	9 (32.1%)	12 (57.1%)	
Class IV	2 (7.1%)	4 (19.0%)	
Class V	0 (0%)	2 (9.5%)	
Hass	(n = 28)	(n = 21)	0.001
Class I	0 (0%)	0 (0%)	
Class II	3 (10.7%)	0 (0%)	
Class III	14 (50.0%)	2 (9.5%)	
Class IV	11 (39.3%)	14 (66.7%)	
Class V	0 (0%)	5 (23.8%)	

### Animal experiments and expression of fibrosis-related markers

**Fig 2:**
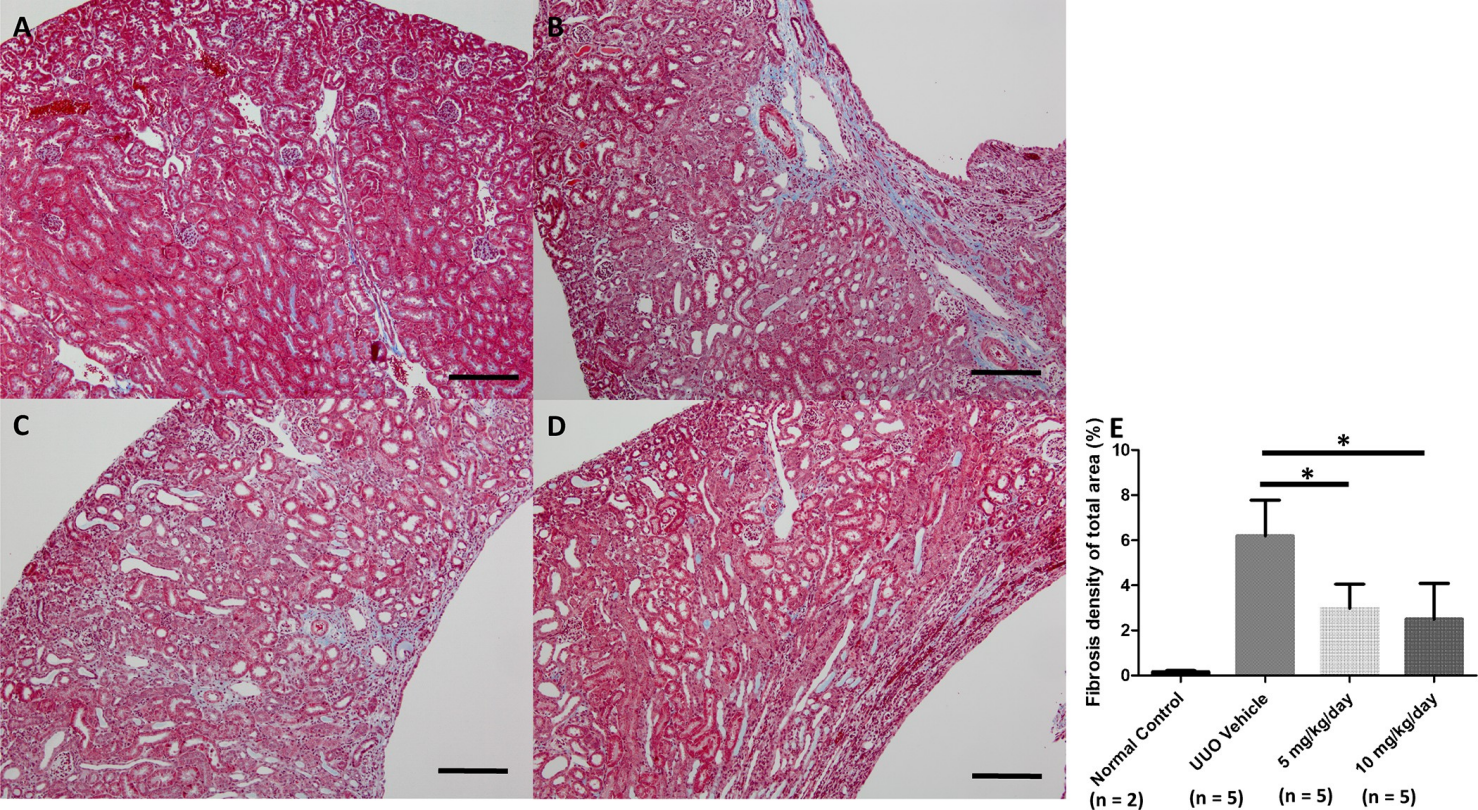
Histologic Masson's trichrome stain findings of renal fibrosis after UUO and administration of a p38 MAPK inhibitor. Scale bar = 100 μm (original magnification x100). Mice from the sham-operated control group (A) show sparse tubulointerstitial fibrosis. UUO-operated group with administered 20% DMSO vehicle (B) shows prominent tubulointerstitial fibrosis. UUO-operated group administered p38 MAPK inhibitor at 5 mg/kg/day (C). UUO-operated group administered p38 MAPK inhibitor at 10 mg/kg/day (D). The extent of tubulointerstitial fibrosis in the groups administered p38 MAPK inhibitor was lower than that in the vehicle group (E). *, *P* < 0.05; UUO, unilateral ureteral obstruction. (n = 5 per group for each experiment, and n = 2 for sham-operated control group).

We performed the UUO operation to induce pathological fibrosis in chronic kidney disease and investigated whether fibrosis was relieved after treatment with a p38 MAPK inhibitor. Interstitial fibrosis was induced in the UUO group but not in the sham-operated control group (Fig 2). The areas of interstitial fibrosis displayed by Masson's trichrome staining were increased in the UUO mice compared to those in the sham-operated group. This degree of fibrosis observed in cortical and medullary lesions was attenuated in p38 MAPK inhibitor-treated UUO mice to a greater extent compared with that in vehicle-treated UUO mice (vehicle vs. 5 and 10 mg/kg/day group, *P* = 0.023, vehicle vs. 10 mg/kg/day group, *P* = 0.029).

**Fig 3:**
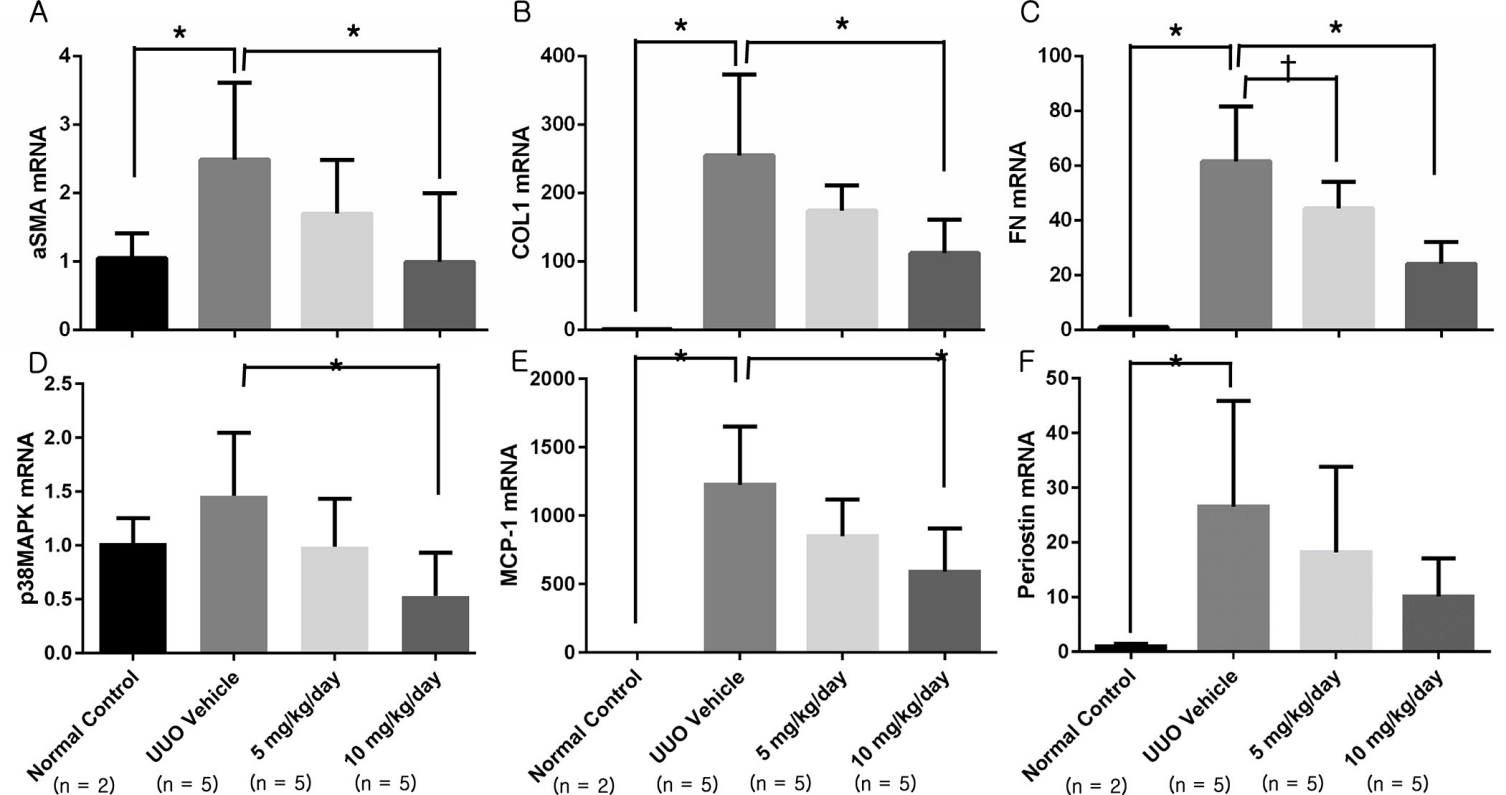
Fibrosis-related mRNA expression as assessed with RT-PCR. (A) Expression of αSMA was decreased in the 10 mg/kg/day p38 MAPK inhibitor group. (B) Expression of COL1 was decreased in the 10 mg/kg/day p38 MAPK inhibitor group. (C) Expression of fibronectin was decreased in the 5 and 10 mg/kg/day p38 MAPK inhibitor groups, with a dose-dependent pattern. (D) Expression of p38 MAPK was decreased in the 10 mg/kg/day p38 MAPK inhibitor group. (E) Expression of MCP-1 was decreased in the 10 mg/kg/day p38 MAPK inhibitor group. (F) The decrease in periostin expression was not statistically significant. αSMA, α-smooth muscle actin; COL1, type 1 collagen; MCP-1, monocyte chemoattractant protein-1; UUO, unilateral ureteral obstruction. *, *P* < 0.01; ^†^, *P* < 0.05. (n = 5 per group for each experiment, and n = 2 for sham-operated control group).

Mice in the UUO group showed a significant increase in the mRNA expression levels of α-smooth muscle actin (αSMA), collagen-1 (COL1), fibronectin, monocyte chemoattractant protein-1 (MCP-1), and periostin (Fig 3). The mRNA expression of fibrosis-related markers, including αSMA, COL1, fibronectin, and MCP-1, decreased significantly after administration of the p38 MAPK inhibitor, and the mRNA expression of fibronectin decreased in a dose-dependent manner. The mRNA expression of periostin increased significantly after UUO, and the mRNA expression of periostin showed a decreasing tendency after p38 MAPK inhibitor administration.

**Fig 4:**
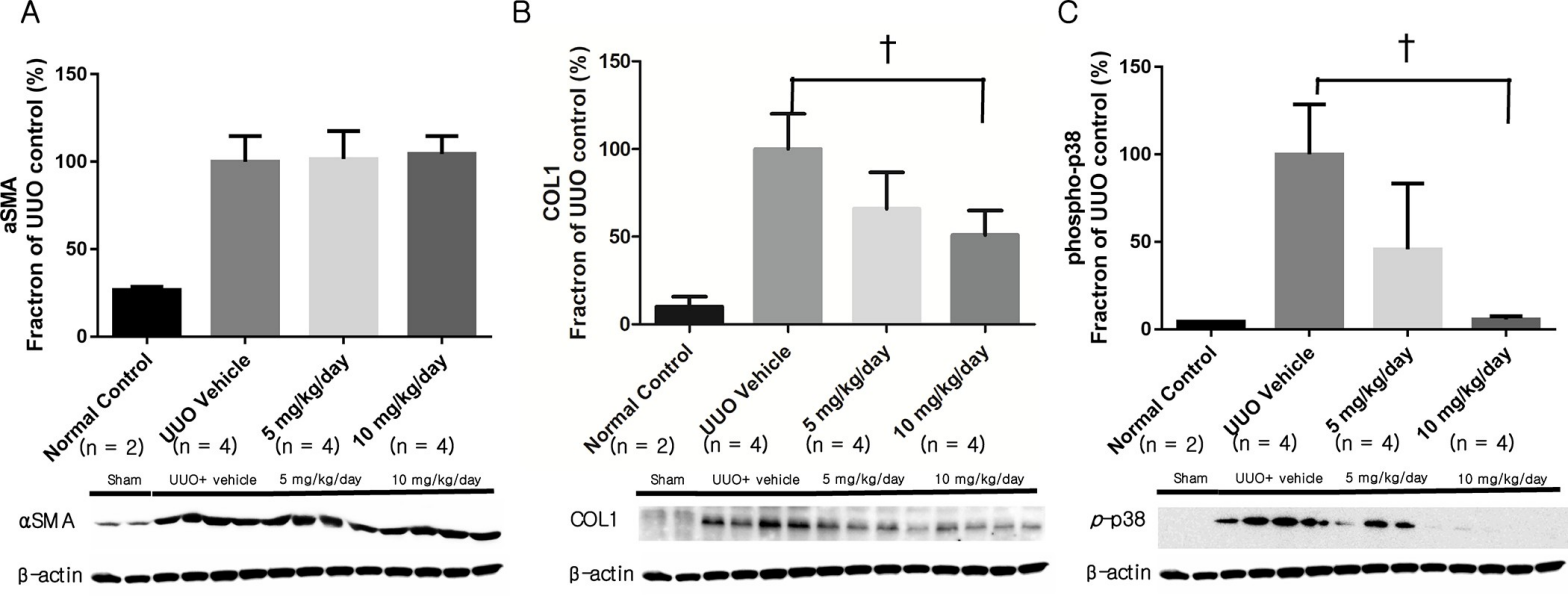
Fibrosis-related renal protein expression as assessed with Western blot. (A) Expression of αSMA did not decrease after p38 MAPK inhibitor administration. (B) Expression of COL1 decreased significantly after administration of 10 mg/kg/day p38 MAPK inhibitor. (C) Expression of phosphorylated p38 MAPK decreased significantly after administration of 10 mg/kg/day p38 MAPK inhibitor. αSMA, α-smooth muscle actin; COL1, type 1 collagen. ^†^, *P* < 0.05. (n = 4 per group for each experiment, and n = 2 for sham-operated control group).

The protein abundance of αSMA, COL1, and phosphorylated p38 in renal tissue was increased in the UUO group (Fig 4). Although the expression of αSMA was not decreased in the groups treated with p38 MAPK inhibitor, the abundance of COL1 and phosphorylated p38 in renal tissue was decreased in the group administered 10 mg/kg/day p38 MAPK inhibitor.

**Fig 5:**
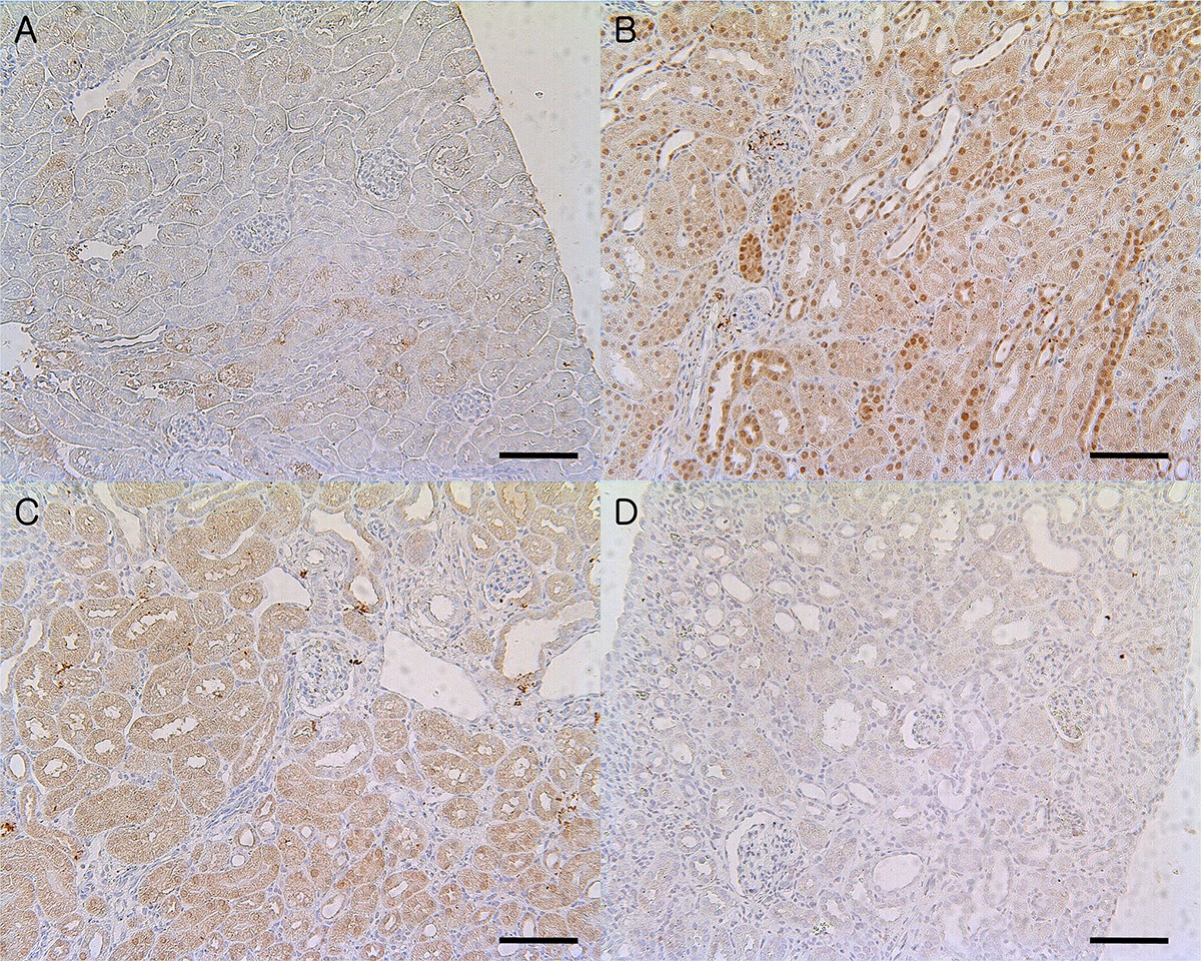
Immunohistochemical staining showing the expression of phosphorylated p38 in the mouse renal tubulointerstitial area after UUO and administration of p38 MAPK inhibitor. Scale bar = 50 μm (original magnification x300). (A) Sham-operated group. (B) UUO-operated group administered 20% DMSO vehicle. (C) UUO-operated group administered 5 mg/kg/day p38 MAPK inhibitor. (D) UUO-operated group administered 10 mg/kg/day p38 MAPK inhibitor. Tubulointerstitial expression of phosphorylated p38 decreased significantly in the groups treated with p38 MAPK inhibitor. UUO, unilateral ureteral obstruction.

We investigated whether p38 MAPK activity was increased in the UUO mouse model and whether a p38 MAPK inhibitor attenuated p38 MAPK kidney tissue activity. We identified the extent of phosphorylated p38 activity in mouse renal tissue using immunohistochemistry methods (Fig 5). The tissue intensity of phosphorylated p38 in the UUO group was significantly increased compared to that in the sham-operated group (*P* = 0.028 sham vs. vehicle group). In the groups with treated with 5 mg/kg/day and 10 mg/kg/day p38 MAPK inhibitor, the level of phosphorylated p38 showed a tendency to decrease compared with that in the vehicle group (S1 Fig, *P* = 0.20 vehicle group vs. p38 MAPK inhibitor 5 mg/kg/day group).

### *In vitro* cellular experiments and expression of fibrosis-related markers

**Fig 6:**
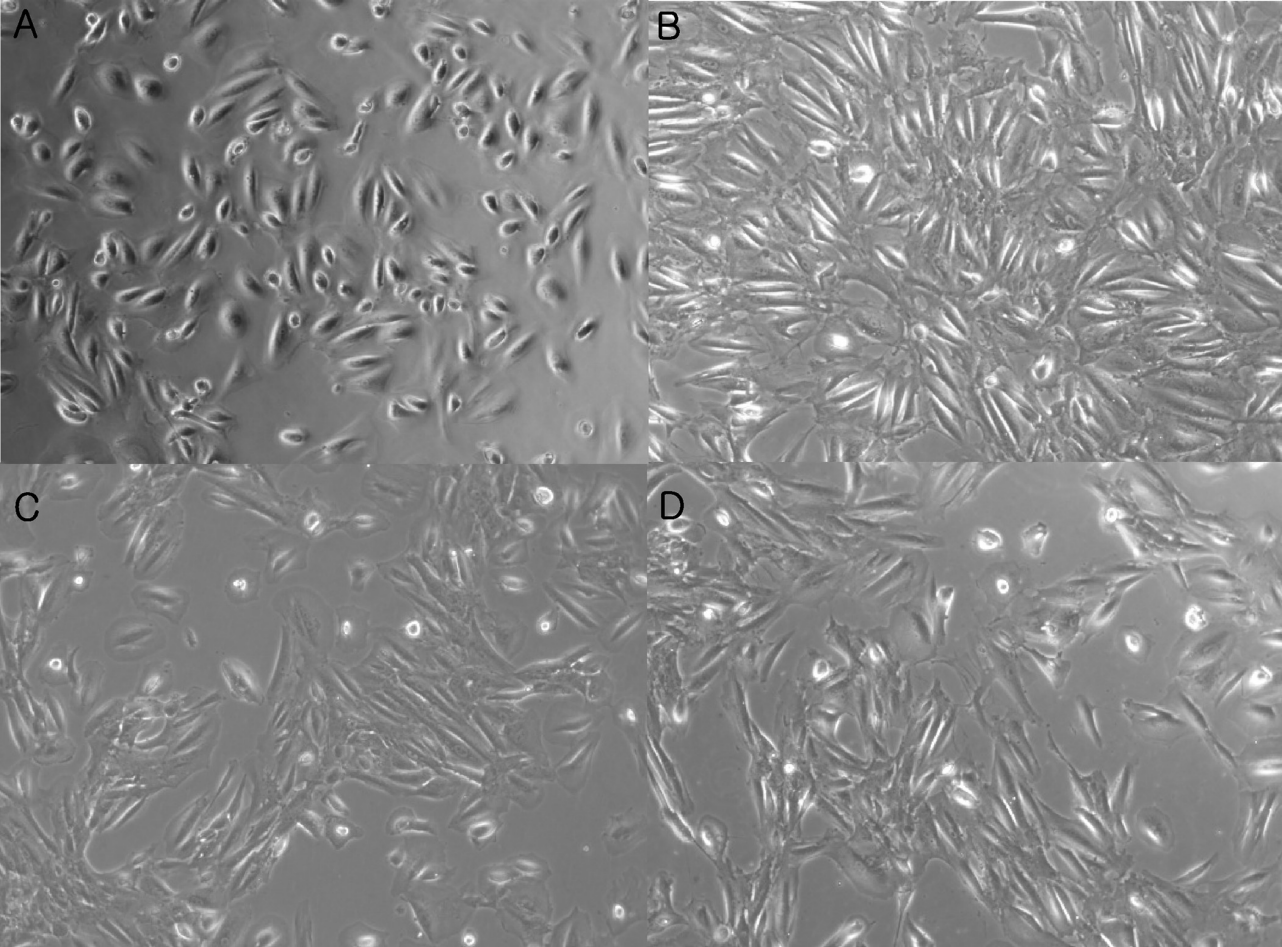
Microscopic morphological changes in hTECs after treatment with p38 MAPK inhibitor. (A) Control hTECs show a normal ellipsoid shape and clear contour. (B) Exposure to TGF-β (2 ng/ml) induced bursting and speckled morphological changes in hTECs. (C) Exposure to TGF-β (2 ng/ml) in the presence of p38 MAPK inhibitor (2 μM). (D) Exposure to TGF-β (2 ng/ml) in the presence of p38 MAPK inhibitor (4 μM).

**Fig 7:**
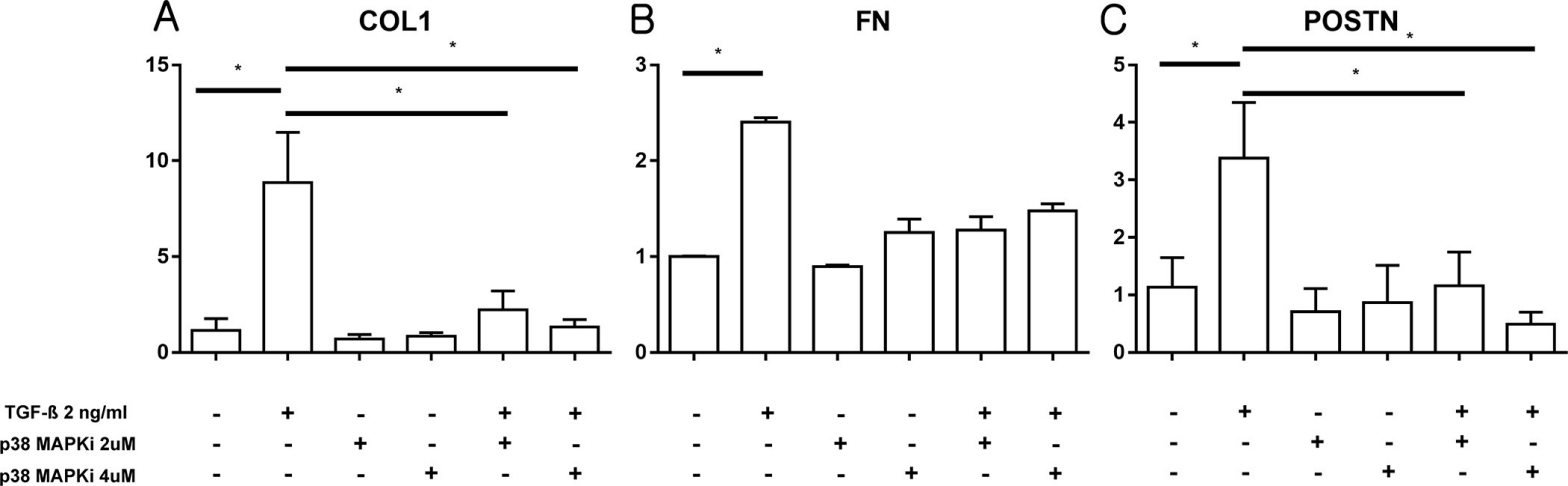
Cellular mRNA expression of fibrosis-related markers after exposure to TGF-β in the presence of p38 MAPK inhibitor. (A) COL1 expression was increased after exposure to TGF-β (2 ng/ml). Treatment with the p38 MAPK inhibitor significantly reduced the cellular expression of COL1. (B) The decrease in the expression of fibronectin was not significant after concomitant exposure to TGF-β and p38 MAPK inhibitor. (C) Expression of periostin was increased after exposure to TGF-β (2 ng/ml). Treatment with p38 MAPK inhibitor reduced TGF-β-induced periostin expression. (n = 6 per group for each experiment, and *in vitro* experiments were repeated 3 times to confirm reproducibility) *, *P* < 0.01.

Laboratory *in vitro* experiments were performed to demonstrate the effect of the p38 MAPK inhibitor. Exposure to TGF-β (2 ng/ml) induced bursting and speckled morphological changes in hTECs compared to the control treatment (Fig 6). Cotreatment with the p38 MAPK inhibitor (2 μM) and TGF-β reversed these cellular morphological changes. The mRNA expression of COL1, fibronectin, and periostin increased after exposure to TGF-β (2 ng/ml) (Fig 7), whereas the treatment with p38 MAPK inhibitor reduced TGF-β-induced COL1 and periostin expression. The decrease in mRNA expression of fibronectin after p38 MAPK inhibitor treatment was not significant (*P* = 0.333).

## Discussion

We found that the increase in immunostaining intensity for phosphorylated p38 MAPK is associated with the degree of renal fibrosis in kidney specimens from IgA nephropathy patients. In addition, we discovered that the immunoactivity of phosphorylated p38 MAPK increases after the development of kidney fibrosis and that a p38 MAPK inhibitor can prevent the molecular, cellular and histological fibrotic response in a UUO mouse model.

Tubulointerstitial fibrosis is the final common pathway for most progressive kidney diseases, leading to advanced chronic kidney disease [20–22]. The histopathological characteristics of tubulointerstitial fibrosis are the deposition of interstitial matrix with inflammatory cells, loss of tubular cells, fibroblast accumulation, and the disappearance of the peritubular microvasculature [23]. Persistent cytokine exposure and the accumulation of inflammatory cells are the principal causes of chronic progressive tubulointerstitial fibrosis [24]. The intracellular signal transduction mediator p38 MAPK plays a functional role in inflammation, apoptosis, and fibrosis [25]. The role of p38 MAPK in inflammation and fibrosis has previously been investigated in models of rheumatoid arthritis, sepsis, and cardiac and peritoneal fibrosis [26–31]. In the kidney, p38 MAPK signaling also contributes to renal inflammation and fibrosis [32]. Therefore, p38 MAPK can be a therapeutic target for preventing the progression of chronic kidney disease.

We established a mouse renal fibrosis model by performing a UUO operation. UUO is a good model of tubular apoptosis and interstitial fibrosis in the kidneys [33]. Mechanical stress induces excessive polymerization of microtubules and contributes to tissue morphogenesis, remodeling and fibrosis [34]. In the UUO fibrosis model, the renal mRNA and protein levels of TGF-β1 increased significantly [17]. In addition, increased p38 MAPK activity (i.e., phosphorylation) was demonstrated in both interstitial myofibroblasts and renal tubules [35]. Activation of p38 and JNK signaling is prominent in tubular epithelial cells in the UUO model of renal fibrosis [36], and renal tubules are the major site of p38 and JNK activation in the obstructed kidney. In our experiments, we investigated whether the activity of phosphorylated p38 is significantly increased in a mouse UUO fibrosis model and whether it is associated with the degree of fibrosis. We observed that kidney tissue activity of phosphorylated p38 MAPK increased mainly in kidney tubules and interstitial areas both in the UUO mouse model and human IgA nephropathy kidney specimens. The increase in kidney tissue phosphorylated p38 activity following UUO was attenuated after p38 MAPK inhibitor administration. After p38 MAPK inhibitor treatment, indicators of fibrosis, including the mRNA expression of αSMA, COL1, and fibronectin, were significantly reduced along with the levels of the inflammatory mediator MCP-1. The mRNA expression of periostin after p38 MAPK inhibition showed only a tendency to decrease. Periostin is known to play an important role in kidney fibrosis [37,38]. This finding might suggest that a p38 MAPK independent pathway involving periostin, such as the TGF-β/Smad pathway, could contribute to the development of kidney fibrosis [39–41].

In the Western blotting analysis, the increased protein expression of phosphorylated p38 decreased along with that of COL1 after p38 MAPK inhibition. Although the mRNA expression of αSMA decreased after p38 MAPK inhibitor treatment, the protein abundance of αSMA did not change. This discrepancy can result from the protein stability and differences in half-life between mRNA and proteins [42]. *In vitro* experiments demonstrated that the p38 MAPK inhibitor successfully prevented the TGF-β-induced fibrosis response in tubular epithelial cells. TGF-β plays a crucial role in the promotion of renal fibrosis, and p38 MAPK inhibition suppresses TGF-β-related fibrosis; this outcome may be consistent with results indicating that treatment with a p38 MAPK inhibitor reduces renal fibrosis [43].

In this study, we investigated whether the degree of fibrosis in the kidneys of patients with IgA nephropathy was associated with the tissue activity of phosphorylated p38. We found that a high degree of histologic fibrosis trended toward high phosphorylated p38 tissue activity based on immunohistochemical staining. In addition, a high degree of phosphorylated p38 activity was significantly associated with a high degree of kidney tissue interstitial fibrosis, inflammation, and tubular atrophy in human tissues. To date, no studies have shown an association between p38 MAPK activity in human kidney and kidney fibrosis. The tissue activity of phosphorylated p38 was not associated with vascular hyalinosis, arteriosclerosis or crescent formation.

Although many studies have shown that the activation of p38 MAPK is related to the development and progression of disease in animal models, few studies have demonstrated that the activity of p38 MAPK in human tissues is related to the disease activity or prognosis. Gaffey et al. showed that phosphorylated p38 expression in lung tissue was increased in patients with chronic obstructive lung disease (COPD) compared to non-COPD controls among lung cancer operation patients using immunohistochemical staining methods [44]. Wang et al. found that phosphorylated p38 expression in breast cancer samples was significantly associated with clinical parameters and pathological profiles of progesterone receptor and proto-oncogene HER2/neu (HER2) [45]. Stambe et al. reported that the p38 MAPK pathway is activated in an anti-glomerular basement membrane disease rat model of crescentic glomerulonephritis and contributes to chronic inflammation and fibrosis [35]. To the best of our knowledge, this study is the first to examine the relationship between the expression of phosphorylated p38 in human kidney tissue and the pathologic status of glomerulonephritis.

Activity of p38 MAPK was previously revealed to be closely associated with disease development and progression in humans and animal experimental models of inflammation and fibrosis. Based on these findings, p38 MAPK has been a therapeutic target for chronic inflammatory or fibrosis disease. Auger-Messier et al. reported that pharmacological inhibition of p38 MAPK using SB-731445 (50 mg/kg in mouse chow) successfully attenuated cardiac fibrosis in a mouse model [12]. Wissing et al. showed that a p38 MAPK inhibitor (SB-731445, 12.5 and 50 mg/kg in mouse chow) prevented myofiber damage and muscular fibrosis in a mouse model of muscular dystrophy [46]. In our animal experiments, we could confirm the antifibrotic effect of a p38 MAPK inhibitor at a dosage of 5 or 10 mg/kg administered via intraperitoneal injection methods. Several studies have been conducted to evaluate the efficacy of a p38 MAPK inhibitor in actual diseases, including rheumatoid arthritis and COPD [47–50]. However, few studies have reported significant effects. There are several possible explanations for the limited efficacy of p38 MAPK inhibitors in clinical studies. Although p38 MAPK is activated in pathological conditions such as inflammation and fibrosis, normal physiological functions are also performed by p38 MAPK. p38 MAPK is required during fetal development of the kidney and is a normal physiological defense mechanism against osmotic stress [51–53]. In fact, side effects of the p38 MAPK inhibitor have been reported in clinical trials, including hepatotoxicity, neurotoxicity, skin rashes, and increased infection susceptibility [54,55]. ASK1, an upstream component of the p38 MAPK pathway, is not activated in the normal physiological state; rather, it is mainly activated in the pathological state. A clinical study is underway to investigate the efficacy of MAPK pathway inhibition for diabetic nephropathy using an ASK1 inhibitor to overcome the disadvantages of p38 MAPK inhibitors [56].

In this study, we demonstrated that the renal tissue activity of p38 MAPK is related to the degree of fibrosis in IgA nephropathy patients and that the inhibitory effect of a p38 MAPK inhibitor can be identified using the immunostaining intensity of phosphorylated p38 in kidney tissue. However, this study has some limitations. There were not sufficient samples from patients with high-grade interstitial fibrosis among the kidney biopsy specimens. Furthermore, only 2 patients had a severe grade of interstitial fibrosis, and the proportion of patients with moderate to severe interstitial fibrosis was only 15%. Therefore, the result of this study on the association of tissue p38 MAPK immunoactivity and the grade of interstitial fibrosis cannot be fully generalized to patients with advanced kidney disease. In addition, patients included in this study had relatively early stage glomerulonephritis with good renal function. Further studies are needed to determine whether phosphorylated p38 activity correlates with the degree of interstitial fibrosis in patients with more severe interstitial fibrosis and a poor glomerular filtration rate.

The p38 MAPK pathway is an intracellular signal transduction pathway involved in the production of proinflammatory and profibrotic mediators, and it is important in the pathogenesis of kidney fibrosis. Activation of p38 MAPK is associated with renal fibrosis, and inhibiting p38 MAPK can attenuate the progression of fibrosis. In this study, we used a mouse UUO model and human IgA nephropathy biopsy specimens and observed that the immunoactivity of phosphorylated p38 is closely associated with renal tubulointerstitial fibrosis and that inhibiting p38 MAPK can prevent the progression of renal fibrosis. Further studies are warranted to prove the efficacy of p38 MAPK inhibition in the prevention of chronic kidney disease in humans.

## Supporting information

S1 Fig Tissue density of phosphorylated p38 after p38 MAPK inhibitor treatment in a mouse UUO model. (TIF)

S1 Table Antibodies used in immunohistochemistry and Western blotting. (DOCX)

S2 Table Clinical characteristics according to the performance of phospho-p38 immunohistochemical staining of kidney biopsy specimens from IgA nephropathy subjects. (DOCX)
