# Supplementary figures and images for: p38 MAPK activity is associated with the histological degree of interstitial fibrosis in IgA nephropathy patients
Source: PLoS One. 2019 Mar 21;14(3):e0213981. doi: 10.1371/journal.pone.0213981 (PMC6428396; doi:10.1371/journal.pone.0213981)

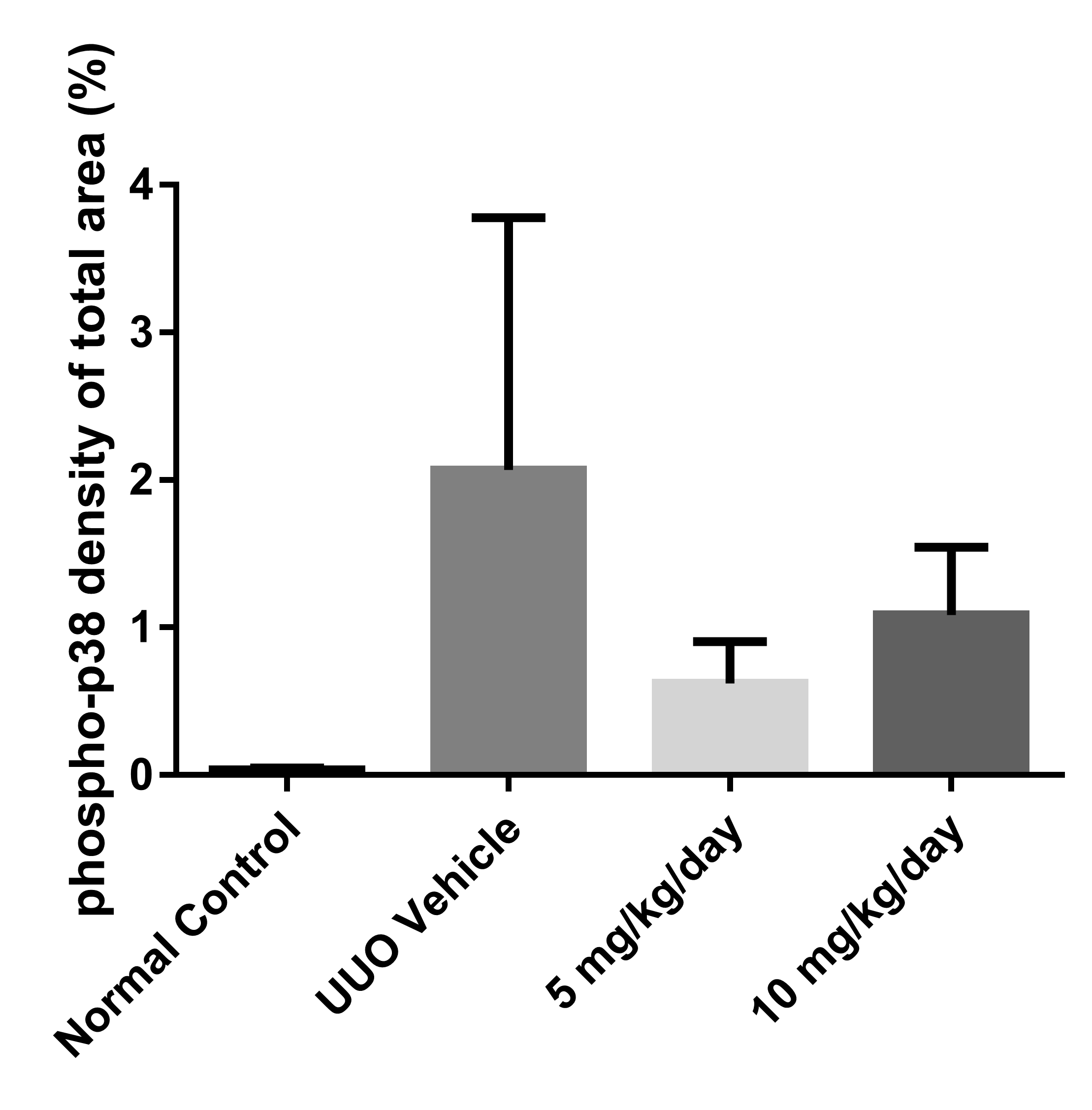

Supplement: S1 Fig — (TIF) [file pone.0213981.s001.tif]
