# Supplementary material for: p38 MAPK activity is associated with the histological degree of interstitial fibrosis in IgA nephropathy patients
Source: PLoS One. 2019 Mar 21;14(3):e0213981. doi: 10.1371/journal.pone.0213981 (PMC6428396; doi:10.1371/journal.pone.0213981)
Supplement: S1 Table — (DOCX) [file pone.0213981.s002.docx]

Supplementary Table 1. Antibodies used in immunohistochemistry and Western blotting

| Antibodies | Vendors |
| --- | --- |
| Anti-p38 MAPK rabbit polyclonal antibody | Abcam, ab7952 |
| Anti-phospho-p38 MAPK (Thr180/Tyr182) rabbit monoclonal antibody | Cell signaling, #9215 |
| Anti-phospho-p38 MAPK alpha (Thr180, Tyr182) rabbit polyclonal antibody | TermoFisher, 44-684G |
| Anti-p53 MAPK rabbit polyclonal antibody | Santa Cruz, SC-6243 |
| Anti-phospho-p53 (Ser15) MAPK rabbit antibody | Cell signaling, #9284 |
| Anti-collagen 1 rabbit polyclonal antibody | Abcam, ab21286 |
| Anti-alpha smooth muscle actin rabbit polyclonal antibody | Abcam, ab5694 |
| Anti-TGF beta rabbit polyclonal antibody | Abcam, ab66043 |
