# Supplementary material for: p38 MAPK activity is associated with the histological degree of interstitial fibrosis in IgA nephropathy patients
Source: PLoS One. 2019 Mar 21;14(3):e0213981. doi: 10.1371/journal.pone.0213981 (PMC6428396; doi:10.1371/journal.pone.0213981)
Supplement: S2 Table — (DOCX) [file pone.0213981.s003.docx]

Supplementary Table 2. Clinical characteristics according to the performance of phospho-p38 immunohistochemical stain on kidney biopsy specimen of IgA nephropathy subjects

| Characteristics | p38 immunohistochemical stain | | *P* value |
| --- | --- | --- | --- |
|  | Immunohistochemical stain not performed  (n = 284) | Immunohistochemical stain performed  (n = 57) |  |
| Age, years | 39.8 ± 15.1 | 42.1 ± 15.7 | 0.304 |
| Gender, male, % | 138 (48.6%) | 32 (56.1%) | 0.298 |
| Systolic BP, mm Hg | 122.1 ± 15.9 | 123.8 ± 14.3 | 0.477 |
| Diastolic BP, mm Hg | 76.9 ± 12.0 | 77.4 ± 10.6 | 0.760 |
| Comorbidity, (%) |  |  |  |
| DM | 13 (4.7%) | 2 (3.6%) | 0.719 |
| HTN | 168 (59.4%) | 31 (55.4%) | 0.578 |
| HBV infection | 8 (3.2%) | 4 (7.1%) | 0.167 |
| HCV infection | 0 (0%) | 1 (1.8%) | 0.180 |
| Serum IgA level, mg/dl | 312.6 ± 107.6 | 294.0 ± 80.5 | 0.240 |
| Serum creatinine, mg/dl | 1.28 ± 1.27 | 1.27 ± 0.47 | 0.991 |
| IDMS-MDRD eGFR, ml/min/1.73 m^2^ | 76.3 ± 32.7 | 75.3 ± 29.0 | 0.985 |
| Serum albumin, g/dl | 3.8 ± 0.5 | 3.9 ± 0.6 | 0.550 |
| Cholesterol, mg/dl | 188.4 ± 39.5 | 185.9 ± 31.6 | 0.660 |
| Uric acid, mg/dl | 6.0 ± 1.7 | 6.3 ± 1.4 | 0.285 |
| hs-CRP, mg/dl | 0.28 ± 0.70 | 0.31 ± 0.58 | 0.823 |
| Spot urine protein/Cr, mg/mg | 1.75 ± 2.34 | 1.78 ± 2.13 | 0.943 |
| Microscopic hematuria | 125/143 (87.4%) | 45/53 (96.3%) | 0.646 |
| Treated with RAS blockade (ACEi/ARB), % | 162 (57.2%) | 34 (60.7%) | 0.631 |
| Treated with statin, % | 53 (32.5%) | 16 (30.2%) | 0.752 |
| Treated with immunosuppressive agents, % | 39/283 (13.8%) | 7/56 (12.5%) | 0.798 |

Abbreviations: Cr, creatinine; BMI, body mass index; BP, blood pressure; HBV, hepatitis B virus; HCV, hepatitis C virus; hs-CRP, high-sensitivity C-reactive protein; RAS, renin-angiotensin-aldosterone system
